# Recent progress in cancer multidrug resistance and therapeutic options associated with protein damage response

**DOI:** 10.1093/procel/pwag032

**Published:** 2026-06-23

**Authors:** Fangyuan Shao, Dongyang Tang, Ling Li, Xiaoling Xu, Chu-Xia Deng

**Affiliations:** Cancer Center, Faculty of Health Sciences, University of Macao, Taipa, Macao 999078, China; Center for Precision Medicine Research and Training, Faculty of Health Sciences, University of Macao, Taipa, Macao 999078, China; MoE Frontiers Science Center for Precision Oncogene, University of Macao, Taipa, Macao 999078, China; Cancer Center, Faculty of Health Sciences, University of Macao, Taipa, Macao 999078, China; Center for Precision Medicine Research and Training, Faculty of Health Sciences, University of Macao, Taipa, Macao 999078, China; Cancer Center, Faculty of Health Sciences, University of Macao, Taipa, Macao 999078, China; Center for Precision Medicine Research and Training, Faculty of Health Sciences, University of Macao, Taipa, Macao 999078, China; Cancer Center, Faculty of Health Sciences, University of Macao, Taipa, Macao 999078, China; Center for Precision Medicine Research and Training, Faculty of Health Sciences, University of Macao, Taipa, Macao 999078, China; MoE Frontiers Science Center for Precision Oncogene, University of Macao, Taipa, Macao 999078, China; University of Macao Advanced Research Institute in Hengqin, Zhuhai 519031, China; Cancer Center, Faculty of Health Sciences, University of Macao, Taipa, Macao 999078, China; Center for Precision Medicine Research and Training, Faculty of Health Sciences, University of Macao, Taipa, Macao 999078, China; MoE Frontiers Science Center for Precision Oncogene, University of Macao, Taipa, Macao 999078, China; University of Macao Advanced Research Institute in Hengqin, Zhuhai 519031, China; Faculty of Synthetic Biology, Shenzhen University of Advanced Technology, Shenzhen 518107, China

**Keywords:** acute drug protein damage, multidrug resistance, protein damage response, mitochondria, mitophagy

## Abstract

Multidrug resistance (MDR) is a common and leading cause of treatment failure and mortality in cancer patients. While numerous biological processes contribute to drug resistance, recent studies have revealed that most anticancer drugs rapidly bind to and damage newly synthesized proteins upon entering cells. This process, termed acute drug protein damage (ADPD), occurs before the drugs act on their canonical targets. To evade the lethal effects of ADPD, cancer cells rapidly initiate a series of protective responses collectively termed the protein damage response (PDR). This cascade includes damage recognition via protein ubiquitination, damage clearance through the proteasome system, and subsequent mitophagy to remove damaged mitochondria caused by the co-import of drugs and damaged proteins. Here, we review the current understanding of multiple biological processes underlying drug resistance, with a focus on the mechanisms of ADPD induced by anticancer drugs, the pivotal role of PDR in driving MDR, and its potential applications in predicting and overcoming drug resistance.

## Introduction

Drug resistance is a common phenomenon in most patients with advanced-stage cancer (Bukowski et al., 2020; Emran et al., 2022). It can arise either from a lack of initial response, known as intrinsic drug resistance, or from a gradual loss of responsiveness during treatment, referred to as acquired drug resistance (Bukowski et al., 2020; Zahreddine and Borden, 2013). Multidrug resistance (MDR) develops when cancer cells become resistant to multiple structurally and mechanistically unrelated drugs, often following sequential or simultaneous exposure to several chemotherapeutic agents (Alexa-Stratulat et al., 2019; Bukowski et al., 2020; Catalano et al., 2022; Ge et al., 2025; Zahreddine and Borden, 2013). Because drug resistance cannot be reliably predicted, it renders tumors refractory to therapy and remains the primary cause of treatment failure and high mortality in patients with late-stage cancer.

MDR is driven by diverse biological mechanisms, including aberrant cell death regulation, cancer stem cell (CSC) activity, cellular drug uptake or excretion, DNA damage and repair, drug metabolism and inactivation, epigenetic regulation, drug target mutation, hypoxia induction, and tumor microenvironment (TME) (Fig. 1). Recent studies by Shao et al. (2025) demonstrated that most of small-molecule anticancer drugs rapidly bind to and damage newly synthesized proteins before reaching their canonical targets by investigating over 100 of them. This damage impairs protein function, particularly of mitochondrial proteins, inducing substantial cytotoxicity. To counteract acute drug protein damage (ADPD), cancer cells rapidly initiate a protective protein damage response (PDR), which involves ubiquitination-mediated damage recognition and proteasome-dependent damage clearance (Shao et al., 2025). Notably, subsequent studies showed that anticancer drugs localized sequentially to mitochondria and lysosomes, suggesting these organelles also participate in the PDR. This is attributed to the fact that 99% of mitochondrial proteins are synthesized in the cytoplasm and remain unfolded prior to mitochondrial import, making them highly susceptible to drug binding and damage (Haastrup et al., 2023; Li et al., 2025; Palmer et al., 2021). The co-import of drugs and damaged proteins impairs mitochondrial function, which, on one hand, activates the proteasome to accelerate damaged protein clearance through reducing ATP production, and on the other hand, triggers mitophagy to eliminate dysfunctional mitochondria. Both processes enable cancer cells to survive anticancer drug-induced cytotoxicity, leading to MDR. In this review, we summarize studies on biological processes associated with MDR, with a focus on PDR, and discuss its potential clinical applications in predicting and overcoming drug resistance to improve cancer therapy outcomes.

**Figure 1.**
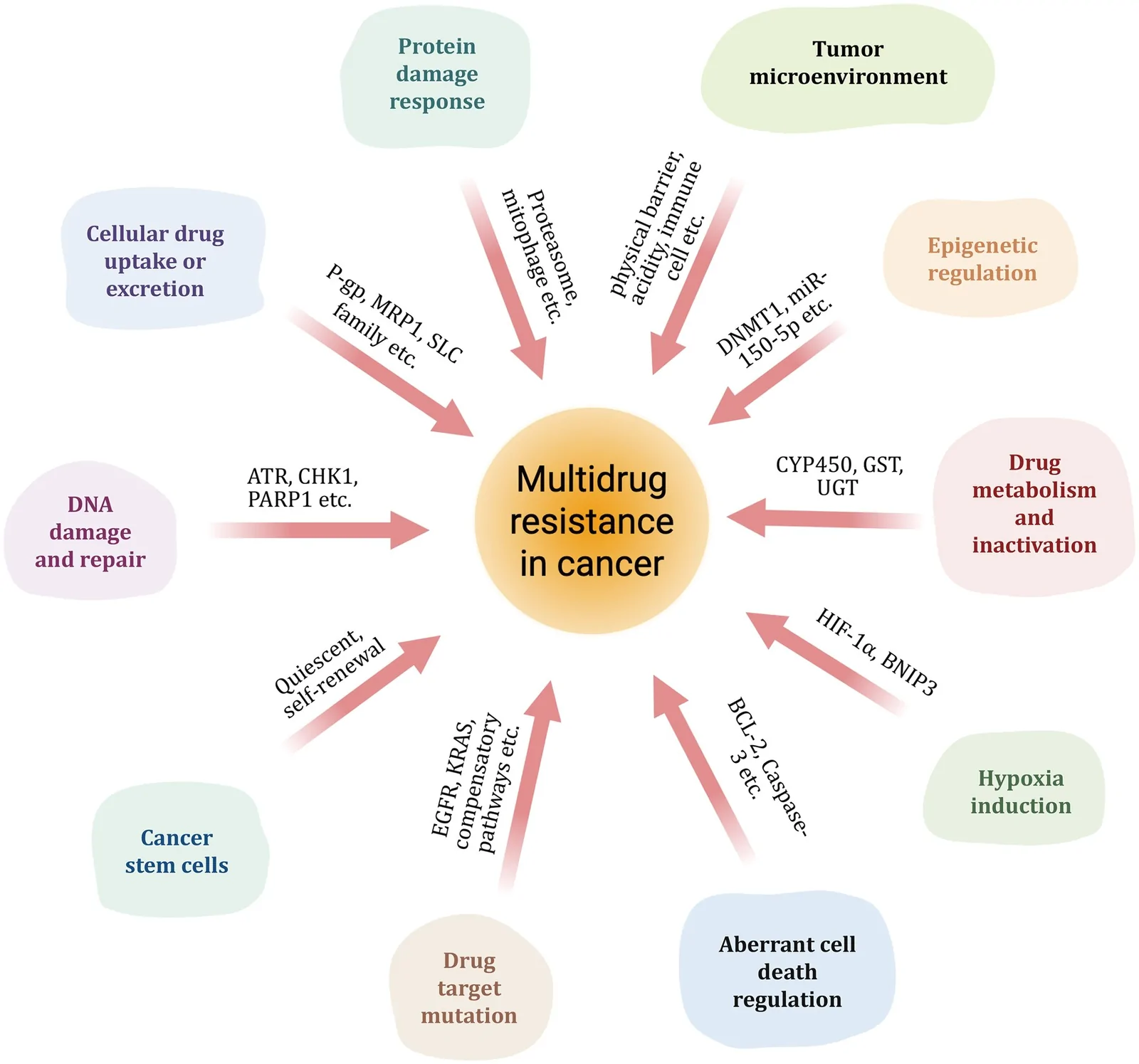
A graphic summary of the main biological processes and key features of the mechanisms leading to MDR.

## Main biological processes involved in cancer drug resistance

Given that many forms of drug resistance (including intrinsic drug resistance, acquired drug resistance, single drug resistance, and multidrug resistance) and their underlying mechanisms have been extensively reviewed previously, we briefly describe their key features, associated biological processes, and therapeutic strategies below.

### Aberrant regulation of cell death

Evasion of cell death, a hallmark of cancer, caused by anti-cancer drugs, is a pivotal mechanism that drives anti-cancer drug resistance. The resistance to intrinsic and extrinsic apoptosis, the most well-characterized programmed cell death (PCD), has been well reviewed (Bou Antoun and Chioni, 2023; Hsu et al., 2025b; Mohammad et al., 2015; Shahar and Larisch, 2020). In addition to apoptosis, cancer cells also developed mechanisms to resist other newly emerged PCD. For example, cancer cells can shut down the expression of key execution protein, RIPK3, in the necroptosis pathway via methylation to prevent the formation of the necrosome complex and therefore the induction of cell death (Preston et al., 2022; Tan et al., 2021), which leads to resistance to necroptosis inducers. Ferroptosis is another type of PCD that relies on iron to trigger lipid peroxidation, membrane damage, and eventually cell rupture (Dixon and Olzmann, 2024). Many anticancer agents can trigger ferroptosis in cancer to exert their antitumor functions. In the context of ferroptosis, it was found that cancer cells could develop intrinsic resistance via the following mechanisms: reducing iron uptake, increasing iron storage, enhancing the function of antioxidant systems, and reducing the production of reactive oxygen and nitrogen species (Lee and Dixon, 2025). As new PCD types are identified, the mechanisms underlying resistance to specific cell death pathways will be further elucidated, providing novel strategies to combat anticancer drug resistance.

Therapeutic strategies can be developed by reactivating cell death pathways using pathway-specific inducers or inhibitors (e.g., BH3 mimetics, SMAC mimetics, ferroptosis inducers, and so on) (Guo et al., 2026).

### Cancer stem cells

Cancer stem cells (CSCs) constitute a small but critical subpopulation within tumors, characterized by self-renewal and the ability to differentiate into diverse cancer cell lineages (Chu et al., 2024; Huang et al., 2020). They are increasingly recognized as key drivers of MDR, contributing to therapeutic failure and tumor relapse. CSCs exhibit intrinsic resistance to conventional chemotherapy and radiotherapy through mechanisms including the quiescent or slow-cycling state, enhanced DNA damage repair, and evasion of apoptosis via elevated levels of anti-apoptotic proteins such as BCL-2 and BCL-XL (Ramesh et al., 2021; Zhou et al., 2017). While these features have been extensively discussed (Lee et al., 2025), this review will summarize additional mechanisms.

The resilience of CSCs is further influenced by dysregulated evolutionarily conserved signaling pathways. Pathways such as Wnt/β-catenin, Hedgehog, Notch, JAK/STAT, and Hippo (via YAP/TAZ) are often hyperactivated, promoting CSC self-renewal, survival, and resistance to various therapies, including cisplatin, radiotherapy, and EGFR inhibitors (Kumar et al., 2021). These pathways contribute to MDR by upregulating *ABC transporters*, activating anti-apoptotic signals, and inducing epithelial-mesenchymal transition (EMT) (Jiang et al., 2025). Furthermore, the tumor microenvironment plays an indispensable role in reinforcing CSC-mediated resistance. Components such as cancer-associated fibroblasts (CAFs) and tumor-associated macrophages (TAMs) secrete cytokines and growth factors (e.g., TGF-β, IL-6) that activate stemness pathways (Gabrusiewicz et al., 2018; Li et al., 2019). Additionally, hypoxic conditions stabilize HIFs, which further promote stem-like and quiescent phenotypes and enhance resistance mechanisms (Emami Nejad et al., 2021; Prasad et al., 2017). The dynamic interaction between CSCs and their niche creates a protective sanctuary that amplifies their ability to withstand therapeutic insults.

Recent evidence suggests that reduced global protein synthesis may represent a novel mechanism through which CSCs acquire multidrug resistance (Aleksandrova et al., 2024; Gupta et al., 2023; Li et al., 2020). CSCs can enter a reversible non-proliferative quiescent state to adapt and survive long-term under therapeutic pressure. This state is characterized by profound suppression of global protein synthesis, enforced through translational repression mechanisms such as eIF2α phosphorylation and mTOR inhibition, which halt canonical cap-dependent translation (Aleksandrova et al., 2024). Moreover, global translational repression enables selective translational reprogramming: alternative pathways mediated by phosphorylated eIF3D facilitate preferential translation of specific mRNAs (many containing upstream open reading frames) encoding proteins that promote survival, stemness maintenance, and a latent aggressive phenotype (Aleksandrova et al., 2024; Gupta et al., 2023).

To effectively eliminate CSCs, a multi-pronged approach is essential, combining direct immune targeting with the disruption of core survival mechanisms. Strategies include using CAR-engineered cells (CAR-T, CAR-NK, CAR-macrophages) to target specific CSC surface antigens such as CD133 and GD2, while simultaneously employing agents (such as small molecules, nanoparticles, and phytochemicals) to inhibit drug efflux pumps, DNA repair pathways, and critical stemness signaling pathways, including Wnt/β-catenin, Notch, and TGF-β (Hadiloo et al., 2025; Jiang et al., 2025). Studies are also directed to uncover new therapeutic opportunities to eradicate CSCs and overcome critical barriers to successful cancer therapy, including ALDH1 inhibition, antibody targeting of CSC markers, inhibition of CSC pathways, i.e., YAP/TAZ, Hedgehog, and JAK/STA (Cho and Kim, 2020; Li et al., 2021).

### Cellular drug uptake or excretion

MDR in cancer can be driven not only by active drug efflux but also by reduced drug uptake. Tumor cells can downregulate solute carrier (SLC) family transporters (e.g., SLC22A1/OCT1, SLC22A2/OCT2), decreasing intracellular accumulation of drugs such as cisplatin, 5‑fluorouracil, and 8‑azaguanine (Anderson et al., 2022; Brosseau and Ramotar, 2019; Girardi et al., 2020; Huttunen and Huttunen, 2022; Schlessinger et al., 2023). Additionally, membrane proteins—including P‑gp—modify the lipid composition, fluidity, and permeability of the plasma membrane in resistant cells, thereby impeding drug entry (Michalak et al., 2006; Taborda Ribas et al., 2024). Membrane proteins can also alter surface charge and membrane potential via calcium channels and sodium pumps, influencing drug ionization and uptake (Huang et al., 2021; Picci et al., 2022).

ATP-binding cassette (ABC) efflux transporters are integral membrane proteins critical for transporting a diverse range of endogenous compounds, including leukotrienes, bile acids, and prostaglandins, as well as xenobiotic substances, notably many chemotherapeutics (Wang et al., 2021b). These transporters, including P-glycoprotein (P-gp/*ABCB1*), Multidrug Resistance-Associated Proteins (MRPs1-9/*ABCCs1-6*, and *ABCCs10-12*) (Kruh and Belinsky, 2003; Li et al., 2018), and Breast Cancer Resistance Protein (BCRP/*ABCG2*), play fundamental roles in cellular drug excretion and are key players in the development of MDR in cancer. Structurally, ABC transporters consist of transmembrane domains (TMDs) forming substrate-binding pockets and nucleotide-binding domains (NBDs) that bind and hydrolyze ATP to drive active efflux (George and Jones, 2012; Wang et al., 2021b). Notably, some MRPs, such as MRP1, possess an additional N-terminal TMD0 domain, categorizing them as “long” MRPs, while others, like MRP4 are “short” and lack this domain (Kroll et al., 2021; Wang et al., 2021b). This structural diversity enables ABC transporters to recognize and extrude a broad spectrum of chemotherapeutic agents from the cytoplasm against concentration gradients, reducing intracellular drug accumulation and compromising therapeutic efficacy.

Many anticancer drugs possess inherent hydrophobicity and lipophilicity, allowing them to penetrate cell membranes and interact non-specifically with critical intracellular components, including cellular membranes, DNA, and various proteins, ultimately leading to cellular damage and toxicity (Alves et al., 2016; Bourgaux and Couvreur, 2014; Li et al., 2025; Shao et al., 2025). These properties make them ideal substrates for ABC transporters, which have evolved to export hydrophobic and xenobiotic compounds. Through specific interactions with transporter substrate-binding pockets, chemotherapeutics are recognized by efflux pumps such as P-gp and BCRP, triggering ATP-driven conformational changes that facilitate active drug extrusion (Engle and Kumar, 2022; Kurimchak et al., 2022). This process reduces intracellular drug concentrations, diminishing chemotherapy efficacy. Targeting ABC transporters to overcome MDR has become a major research focus, with strategies evolving through multiple generations of small-molecule inhibitors (Engle and Kumar, 2022; Jaramillo et al., 2018). However, challenges remain, including toxicity, compensatory mechanisms, and the need for biomarker-driven patient selection. Emerging strategies, including RNA interference and CRISPR/Cas9 gene editing, alongside recent structural insights from cryo-EM studies, offer promising avenues for the rational design of selective inhibitors (Robey et al., 2018; Shirk et al., 2023). Ultimately, a comprehensive strategy integrating functional imaging, biomarker-based stratification, and rational combination therapies is essential for effective clinical translation.

Therapeutic strategies for efflux-related resistance include the use of efflux inhibitors (such as verapamil, cyclosporine A, tariquidar); the use of ABC transporter inhibitors (such as verapamil, elacridar) alongside chemotherapy, and genetically or epigenetically modulated (Bugde et al., 2017; Chen et al., 2016; Pote and Gacche, 2023). On the other hand, the resistance related to drug uptake is more difficult. Targeting membrane protein–induced changes represents a potential strategy to improve drug penetration, and the use of prodrugs that enter via alternative routes or bypassing the need for transporters could also be an alternative (Yang et al., 2024).

### DNA damage and repair

The clinical chemotherapy drugs, including alkylating agents, antibiotics, antimetabolites, topoisomerase inhibitors, and DNA damage repair (DDR) inhibitors through inducing DNA inter-strand or intra-strand crosslinks, interfere with DNA replication, induce DNA strand breaks, and interfere with DDR to generate DNA-damage (Ma et al., 2025). DNA repair is a key mechanism of drug resistance: chemotherapeutics exert antitumor effects by directly or indirectly damaging cancer cell DNA, but cancer cells often utilize DNA repair mechanisms to counteract this damage. For example, platinum-based drugs, like cisplatin, induce DNA damage that triggers tumor cell apoptosis (Basu and Krishnamurthy, 2010; Vassilopoulos et al., 2014; Wang et al., 2021a). The resistance to such agents arises from DNA repair systems, including nucleotide excision repair (NER) and homologous recombination repair mechanisms (HRR), which restore the integrity of damaged DNA in cancer cells (Huang and Zhou, 2021; Jurkovicova et al., 2022). Consequently, the effectiveness of these agents hinges on the ability to inhibit DNA repair processes within the cancer cells. Suppressing DNA repair functions enhances the sensitivity of cancer cells to these drugs, thereby boosting the efficacy of chemotherapy. Furthermore, targeting defects in DNA repair systems, which may result from mutations or epigenetic silencing within cancer cells, stands as a potential therapeutic strategy (Hopkins et al., 2022). Enhanced DNA repair activity and alkyltransferase function have also been linked to resistance against alkylating agents such as doxorubicin (de Pagter and Kloosterman, 2015).

Therapeutic strategies include the use of inhibitors for DNA repair kinases to overcome resistance associated with DNA-damaging drugs, such as doxorubicin and cisplatin, in breast cancer cells (Stefanski et al., 2019; Xu et al., 2019). In particular, PARP inhibition is a powerful approach for synthetic lethality with DNA damage-deficient breast cancer-associated gene 1 and 2 (*BRCA1*/*2*) (Byrum et al., 2019; Li and Zou, 2024; Xu et al., 1999). In recent years, immunotherapy has emerged as a major breakthrough in cancer treatment, prompting increased interest in the role of DDR in tumor immunotherapy. Studies indicate that deficiencies in certain DNA repair pathways are associated with improved responses to immune checkpoint blockade (ICB) therapies, highlighting the crosstalk between DDR and antitumor immunity (He et al., 2022; Sun et al., 2021; Zhang et al., 2020).

### Drug metabolism and inactivation

Drug metabolism plays a critical role in the development of drug resistance, as cells or organisms may modify their metabolic enzymes to rapidly inactivate drugs, hinder the activation of prodrugs, or adjust their metabolic pathways to circumvent the therapeutic effects of the drugs. Drug-metabolizing enzymes (DMEs) are one of these factors, for which less study has been conducted. DMEs include Phase I and Phase II enzymes (Kaur et al., 2020). Cytochrome P450s (CYPs) are major phase I enzymes and glutathione-S-transferases (GSTs) (Durairaj and Liu, 2025), and play a role in over 90% of reported enzymatic reactions. In the context of drug metabolism, they are recognized as the most prominent drug-metabolizing enzymes (Pathania et al., 2018). While primarily expressed in the liver, CYPs are also found in other organs including the kidney, placenta, adrenal gland, skin, and gastrointestinal tract (Danielson, 2002). UDP-glucuronosyltransferases (UGTs) and dihydropyrimidine dehydrogenases are the major enzymes belonging to the Phase II enzymes (Pathania et al., 2018). UGTs promote chemoresistance by deactivating drugs through glucuronide conjugation. Clinical research has identified elevated levels of UGT expression in non-responders compared to responders undergoing cytarabine and ribavirin therapy for AML, erlotinib treatment for NSCLC, tamoxifen for breast cancer, and, notably, anti-PD-1 antibody treatment for metastatic renal cell carcinoma (mRCC) (Allain et al., 2020; Pathania et al., 2018).

Drug inactivation is a mechanism employed by bacteria to neutralize drugs, either by eliminating a chemical group from the drug or by transferring it to a different chemical group. *In vivo* interactions between these drugs and various protein types can modify the drugs’ molecular properties, ultimately leading to their activation (Reygaert, 2018). However, cancer cells may develop resistance by decreasing the efficacy of these drugs. Acute myeloid leukemia (AML) treatment with cytarabine (AraC), an anti-cancer drug, relies on its transformation into cytarabine triphosphate (AraC-triphosphate) through multiple phosphorylation steps. Initially, AraC itself has no impact on cancer cells, but once phosphorylated, the resulting compound becomes toxic and damages the cells (Bukowski et al., 2020). However, down-regulation or mutations in the proteins and enzymes responsible for these phosphorylation reactions can diminish AraC’s effectiveness, leading to the development of drug-resistant cancer cells (Michael and Doherty, 2005).

Therapeutic strategies include (i) direct blockade of the upregulated enzymes using specific enzyme inhibitors, (ii) prodrug design to create drugs activated only in the tumors (e.g., capecitabine activated by thymidine phosphorylase in cancer cells), and (iii) deliver agents to destinated sites via protected manner (Giang et al., 2014; Ramsay and Tipton, 2017; Sabit et al., 2025).

### Drug target mutation

Drug target mutations are a key driver of anticancer drug resistance—particularly for targeted therapies. Mutations in canonical drug targets can alter drug binding affinity, modify protein function, or even reverse drug effects, resulting in treatment failure (Huang et al., 2023; Waarts et al., 2022). Epidermal growth factor receptor (EGFR) is a member of the receptor tyrosine kinase (RTK) family and is often mutated in multiple common solid tumors and involved in regulating of cell growth and differentiation (Du et al., 2025; Li et al., 2024a). Different generations of inhibitors targeting EGFR have been developed and approved for use in clinical settings, but resistance remains. For the first and second generation of tyrosine kinase inhibitors (TKIs) like erlotinib and gefitinib, it was found that patients can develop a T790M mutation in *EGFR*, which will lead to changes in conformation of EGFR and impair the binding of these TKIs to EGFR (Chan and Hughes, 2014). To tackle this clinical need, the third generation of TKIs, including Osimertinib and Rociletinib, was developed. Although these drugs are able to reverse the drug resistance to the first and second generation of TKIs caused by T790M, unfortunately, it is found that cancers can gain a C797S mutation, which can mediate the resistance to osimertinib and rociletinib (Yang et al., 2018). Therefore, the fourth generation of TKIs that are sensitive to EGFR with T790M and C797S mutation was designed and is now undergoing clinical trial (Corvaja et al., 2024). Recent studies also highlight the persistent challenges in targeting Kirsten rat sarcoma viral oncogene homolog (KRAS) mutations, one of the most common oncogenic drivers in pancreatic, colorectal, and lung cancers. While KRAS G12C inhibitors such as sotorasib and adagrasib have achieved modest clinical success, particularly in non-small cell lung cancer, their efficacy remains limited by the rapid emergence of resistance through secondary mutations. For example, the *de novo* mutations, including G13D, A59S, A59T, R68M, Y96D, and Y96M, alter the binding ability of KRAS G12C inhibitors to these oncogenic proteins (Awad Mark et al., 2021; Koga et al., 2021). The emergence of new EGFR and KRAS mutations with existing specific inhibitors advanced our understanding of resistance to target therapy drugs and the development of novel therapeutic strategies (Choucair et al., 2025; Naykwadi and Alavala, 2026; Wang et al., 2025).

Activation of some other pathways also contributes to the drug resistance development. A CRISPR/Cas9-based resistance screen revealed that components of the DNA mismatch repair pathway contribute to resistance to 6-thioguanine. The screen further demonstrated that loss of *TOP2A* confers strong protection against etoposide and identified CDK6 as a critical mediator of drug-induced cytotoxicity (Wang et al., 2014). Another genome-wide CRISPR KO screen carried out in MCF-7 cells developing resistance to different ER inhibitors uncovered the role of purine biosynthesis pathways (Hany et al., 2023). Screening in NSCLC HCC287 cells uncovered key determinants of resistance to the EGFR TKI erlotinib, implicating the YAP signaling pathway as a negative regulator of resistance and the cullin-5 E3 ubiquitin ligase complex as a positive contributor (Zeng et al., 2019). In erlotinib-resistant NCI-H820 lung cancer cells, gene signatures associated with the cell division cycle and protein ubiquitination were identified, and targeting either pathway led to similarly increased antitumor efficacy *in vivo* (Lee et al., 2021).

### Epigenetic modification caused drug resistance

Epigenetic modifications are increasingly recognized as fundamental regulators of cancer biology (Sadida et al., 2024), with accumulating evidence indicating that aberrant epigenetic mechanisms—including DNA methylation, histone modifications, and non-coding RNA (ncRNA) regulation—drive tumor resistance (Adhikari et al., 2022; Liu et al., 2020; Wang et al., 2021c). DNA methylation represents a key epigenetic mechanism characterized by the addition of a methyl group to the cytosine residue, specifically at the 5′ carbon within CpG islands, typically situated upstream of promoter regions. While also observed at various other loci across the genome (Moore et al., 2013).

Furthermore, acetylation and deacetylation of lysine residues located at the terminal ends of histones and certain non-histone proteins are mediated by histone acetyltransferases (HATs) and histone deacetylases (HDACs), respectively. These enzymatic activities play a critical role in regulating chromatin structure and gene expression (Lee et al., 2020). Tumor suppressor genes are frequently silenced through methylation processes. The demethylation of the multidrug resistance gene (*MDR1*) in cancer cell lines results in the development of multidrug resistance (Sharma and Vertino, 2004). Baharudin et al. previously conducted DNA methylation profiling on 5 recurrent and 43 non-recurrent colorectal cancer (CRC) patients undergoing treatment with 5-fluorouracil (5-FU). Their analysis revealed 4,787 genes with significant differential methylation between the recurrent and non-recurrent groups. Among these, 3,112 genes exhibited hypermethylation, while 1,675 displayed hypomethylation (Baharudin et al., 2017). Emerging reports demonstrated that these modifications can lead to significant downstream effects, such as the activation of oncogenic signaling pathways, the preservation of CSC features, the initiation of EMT, and the inhibition of apoptosis. DNA methyltransferases like DNMT1 and DNMT3 are strongly implicated in mediating resistance to drugs such as cisplatin, tamoxifen, and daunorubicin (Hervouet et al., 2013; Wang et al., 2021c).

At the same time, miRNAs are small non-coding RNAs that play critical roles in regulating complex biological functions by targeting virtually all genes in the genome, constituting an important epigenetic mechanism (He et al., 2020). The dysregulation of miRNAs has been implicated in the development of cancer and anti-cancer drug resistance (He et al., 2020). Due to its ability to directly modulate gene expression post-transcriptionally, miRNA was deeply involved in the regulation of biological processes, including stemness maintenance, drug influx and efflux, drug metabolism, and cell death signal pathway regulation, and therefore participates in the regulation of anti-cancer drug resistance. Specific miRNA, for example, miR-150-5p, was recently identified as a negative regulator of SLC7A11, a key ferroptosis inhibitor (Zhai et al., 2025). miR-150-5p was revealed to be decreased in expression in tumor tissues, which results in the increase of SLC7A11 expression to rewire the ferroptosis signal cascade and mediate resistance to ferroptosis inducers (Zhai et al., 2025). Lee et al. (2017) identified miR-200a-3p targets dual-specificity phosphatase 6 to mediate the resistance of hepatocellular carcinoma to the treatment of 5-FU, doxorubicin, and cisplatin.

The most common therapeutic strategies to overcome epigenetic-driven drug resistance include the use of epigenetic drugs (Epi-drugs), such as DNA methyltransferase inhibitors, histone deacetylase inhibitors, and histone methyltransferase inhibitors, such as EZH2 inhibitors. Because Epi-drugs restore epigenetic balance and sensitize cells to chemotherapy or targeted therapy, combination therapies of Epi-drug + conventional therapy (such as DNMTi + cisplatin), and Epi-drug + targeted therapy (such as HDACi + tyrosine kinase inhibitors) are used to overcome acquired resistance (Diyabalanage et al., 2013; Suraweera et al., 2025).

### Hypoxia induction

Hypoxia, a common feature of the solid tumor microenvironment, plays a pivotal role in driving MDR. Uncontrolled tumor cell proliferation and dysfunctional vasculature lead to significantly reduced oxygen levels, inducing metabolic stress and stabilizing HIFs (particularly HIF-1α and HIF-2α) (Keith et al., 2011; Ortmann, 2024). These transcription factors orchestrate extensive genetic and metabolic reprogramming, contributing to MDR through mechanisms such as drug efflux, survival pathway activation, metabolic adaptation, and microenvironmental remodeling—collectively enabling tumor cells to evade cytotoxicity from chemotherapy, targeted therapy, and radiation (Chen et al., 2023; Yong et al., 2022).

Under hypoxic conditions, the expression and activity of *ABC transporters*, including P-gp, MRP1, and BCRP, are markedly enhanced (He et al., 2019). HIF-1α directly interacts with hypoxia response elements in the promoters of these genes, resulting in increased drug efflux and diminished intracellular concentrations of chemotherapeutic agents. Concurrently, hypoxia promotes protective autophagy through HIF-mediated upregulation of *BNIP3* and activation of AMPK-ULK1, allowing cells to clear away and recycle damaged components while alleviating therapy-induced injury (Park et al., 2013). Moreover, HIF-1α alters the balance of apoptosis regulators by upregulating anti-apoptotic proteins such as Bcl-2 and Bcl-xL while downregulating pro-apoptotic factors like Bax and Bak, thus effectively increasing the threshold for cell death (Chen et al., 2009).

Hypoxia induces a slow-cycling state in cancer cells, enriching a quiescent population that contributes to tumor recurrence and innate resistance (Emami Nejad et al., 2021; Shang et al., 2025). This hypoxia-induced quiescence promotes the enrichment of CSCs through the upregulation of pluripotency factors such as *OCT4* and *NANOG* (Prasad et al., 2017). Characterized by significantly suppressed global mRNA-to-protein synthesis, this slow-cycling state arises primarily from limited ATP availability due to impaired oxidative phosphorylation (Chee et al., 2019). Despite this global translational repression, cancer cells maintain the production of crucial survival and resistance proteins via a specialized, HIF-2α-mediated pathway (Uniacke et al., 2012). By forming a complex with RBM4 and eIF4E2, HIF-2α directs the selective translation of mRNAs containing ribosomal hypoxia-response elements, ensuring the synthesis of pro-survival factors even when overall protein synthesis is compromised (Staudacher et al., 2015; Uniacke et al., 2012). This adaptive translational rewiring highlights the dual role of HIFs in both transcriptional and translational control of MDR.

Therapeutic strategies to overcome hypoxia-induced resistance include the use of HIF inhibitors, hypoxia-activated prodrugs, vascular normalization, and targeting downstream effectors (e.g., P-gp inhibitors, BCL-2 inhibitors), etc. (Li et al., 2017; Lv et al., 2015; Mendoza et al., 2025).

### PDR and MDR

Cisplatin is a platinum drug that alkylates DNA through the formation of platinum-DNA adducts, which consequently results in DNA damage and cell cycle arrest, leading to apoptosis (Galluzzi et al., 2012; Shen et al., 2012; Vassilopoulos et al., 2014). We previously conducted an siRNA-mediated screening using a custom library containing 56 candidate genes and found that cisplatin resistance was linked to the enrichment of cells with higher levels of *ATP7A*, an efflux transporter that binds to cisplatin and pumps it out of cells, preventing its entry into the nucleus (Chisholm et al., 2016). Further study using a larger siRNA library targeting 704 kinases, we showed that cisplatin activates the ATR-CHK1-WEE1 signaling pathway, halting DNA replication to arrest cells in the S phase—providing additional time for DNA repair and reducing cisplatin-induced lethality (Zheng et al., 2017). To perform an unbiased screening for candidate genes associated with cisplatin resistance, we used a whole-genome library containing 64,755 siRNAs for 21,585 genes, followed by rigorous validation (Shao et al., 2020). The data revealed that siRNA-mediated knockdown of 45 proteasome-related genes sensitized MDA-MB-231 breast cancer cells to cisplatin, while knockdown of 104 mitochondrial respiratory chain-related genes increased drug resistance (Shao et al., 2020). Consistently, a multidrug-resistant cell line, 231-R3, generated via long-term selection with gradually increasing cisplatin concentrations, exhibited higher proteasome activity and lower expression levels of mitochondrial respiratory chain-related genes. Further studies revealed: (i) many anticancer drugs could cause protein damage, resulting in strong cytotoxicity; (ii) to fight with the lethal effect, cancer cells initiate a protein damage response (PDR) characterized by ubiquitination-mediated protein damage recognition and proteasome-dependent clearance of damaged proteins; (iii) the damaged proteins could be imported into the mitochondrion, which impairs mitochondrial respiratory activity and reduces ATP production, leading to the activation of proteasome activity; and (iv) the damaged mitochondria are subsequently removed by ROS-BNIP3-mediated mitophagy. Altogether, these actions enable MDR of cancer cells (Li et al., 2025; Shao et al., 2020, 2025).

To develop effective therapeutic strategies to overcome PDR-mediated drug resistance, researchers have developed a highly sensitive kit for detecting proteasome activity so that cancer patients with higher levels of proteasome activity can be selected (Shao et al., 2025). The use of a proteasome inhibitor in combination with conventional chemotherapy (Tang et al., 2025a) and low-dose irradiation (Shao et al., 2026) has also been developed and tested in multiple model systems.

### Tumor microenvironment and drug resistance

Tumor microenvironment (TME) refers a tumor ecosystem composed of various components including cancer cells, fibroblast cells, immune cells, extracellular matrix (ECM), blood/lymphatic vessels, and numerous signaling molecules (i.e., growth factors, chemokines, metabolites, and exosomes) (Dysthe and Parihar, 2020; Gu and Muller, 2025; Guo and Deng, 2018; Waldner and Neurath, 2023; Zessner-Spitzenberg et al., 2019). While most studies mentioned above emphasize the primary role of genetic mutations and epigenetic alterations of cancer cells in drug resistance, recent data revealed critical roles of most components of TME in mediating cancer growth, drug resistance to chemotherapy, targeted therapy, and immunotherapy (Dysthe and Parihar, 2020; Gu and Muller, 2025; Guo and Deng, 2018; Waldner and Neurath, 2023; Wickramasekara et al., 2026; Zessner-Spitzenberg et al., 2019).

One of the key mechanisms of TME-mediated drug resistance involves a physical barrier and altered drug availability (Hirata et al., 2026). In liver cancer patients, enhanced deposition and cross-linking of type I collagen are frequently detected, which facilitates tumor growth and metastasis by EMT. The collagen enrichment could induce 5-fluorouracil (5FU) and sorafenib resistance in liver cancer cells through hypoxia-induced up-regulation of lysyl oxidase-like 2 (LOXL2) (Gong et al., 2022). Consistently, the inhibition of LOXL2 in a collagen-enriched microenvironment synergistically promotes the efficacy of sorafenib and 5FU through deterioration of focal adhesion signaling (Gong et al., 2022).

Acidic TME (pH 5.6–7.0) is another key driver of cancer drug resistance. It can drive cancer cell migration toward blood vessels that have a relatively higher pH (7.35–7.45), while also hampering immune cell activity to promote tumor progression (Boedtkjer and Pedersen, 2020). The acidic TME is believed to be caused by lactate/H^+^ from glycolysis, bicarbonate, and protons generated from the hydration of CO_2_, as well as the transport of H^+^ from the cytosol to the interstitial space (Le Floch et al., 2011; Stock and Pedersen, 2017). Studying *BRCA1*-mutant mammary tumor model and human breast cancers, Shu et al. identified a distinct mechanism mediated by sialyltransferases that induces an acidic tumor-permissive microenvironment (ATPME). They further showed that the ATPME has increased PD-L1 and PD1 expressions, which render the tumor resistant to anti-PD1 treatment. The use of sialyltransferase inhibitor 3Fax-Peracetyl Neu5Ac could neutralize the ATPME and sensitize cancers to immune checkpoint blockade by activating CD8 T cells, thereby inhibiting tumor growth and metastasis (Shu et al., 2023).

Many signaling molecules in TME also participate in the development of drug resistance, such as EGF, IL-10, S100A9, and TGF-β (Li et al., 2022; Taniguchi et al., 2020; Wei et al., 2026; Zhao et al., 2018). Through comprehensive multi-omics analyses of cancer patients and mouse models, combined with functional validation, Wang et al. (2026) demonstrated that platinum treatment upregulated LARS2 via a danger-triggered host response during resistant tumor progression, concomitant with increased chromatin accessibility. This signaling induces drug resistance through two key mechanisms: enrichment of cancer stem cells and promotion of TGF-beta-mediated immunosuppression, as evidenced by M2 macrophage polarization and CD8+, T cell exhaustion. They also developed an effective therapeutic strategy combining carboplatin with LARS2 signaling pathway inhibition, which successfully reversed platinum resistance and restored PD-1 checkpoint blockade sensitivity in preclinical models (Wang et al., 2026).

Many cellular components of TME also play important roles in the formation of drug resistance. These include myeloid-derived suppressor cells (MDSCs) (Dash et al., 2026), which create an immunosuppressive milieu, reducing the efficacy of immunotherapies and some chemotherapies; Regulatory T cells (Tregs) (Bahrami et al., 2019), which suppress cytotoxic T cell activity, contributing to resistance to checkpoint inhibitors; and cancer-associated fibroblasts (CAFs) (Chen et al., 2025a), which release exosomes containing miRNAs or even chemotherapeutics to confer resistance to adjacent cancer cells.

Therapeutic strategies targeting TME components, reversing immunosuppression, targeting the PH axis, disrupting CAF-cancer crosstalk, blocking integrin/adhesion, and immune checkpoint inhibitors have been developed to fight against cancer drug resistance (Chen et al., 2025a; Cives-Losada et al., 2026; Li et al., 2024b, 2024c; Wickramasekara et al., 2026).

While these main biological processes/mechanisms for drug resistance are briefly described in the text, we also summarize the key features associated with MDR and therapeutic strategies in Table 1 for facilitating the follow-up.

**Table 1.** Overview of major biological mechanisms underlying multidrug resistance and associated therapeutic strategies.

Biological processes involved in MDR	Key features of drug resistance	Therapeutic strategies	**Key references**
**Aberrant cell death regulation**	Dysregulation of cell death pathways (e.g., *Bcl-2* overexpression, caspase inactivation) promotes cell survival, resistance to programmed cell death (necroptosis, ferroptosis, etc.).	Reactivate the cell death pathway by using BH3 mimetics, SMAC mimetics, and so on.	Mohammad et al., 2015, Shahar et al., 2020, Guo et al., 2026
**Cancer stem cells**	Quiescent status, enhanced DNA repair, drug efflux, and self-renewal capacity contribute to therapy resistance and tumor relapse.	ALDH1 inhibition; antibody targeting of CSC markers; inhibition of CSC pathways, i.e., YAP/TAZ, Wnt/β-catenin, Hedgehog, notch signaling, and JAK/STA.	Lee et al., 2025, Jiang et al., 2025, Hadiloo et al., 2025, Li et al., 2021, Cho et al., 2020
**Cellular drug uptake or excretion**	Either reduced drug uptake or enhanced drug efflux, could decrease intracellular drug accumulation and efficacy.	Three generations of ABC transporter inhibitors; tyrosine kinase inhibitors as chemosensitizers; RNAi and CRISPR/Cas9; epigenetic modulation.	Wang et al., 2021, Engle et al., 2022, Kurimchak et al., 2022, Yang et al., 2024
**DNA damage and repair**	Enhanced DNA repair mechanisms (e.g., NER, HR) counteract drug-induced DNA damage; DNA damage response pathways confer resistance.	Combine the DNA damage response inhibitors (ATR, CHK1, WEE1, etc.) with chemotherapy drugs.	Huang et al., 2021, Jurkovicova et al., 2022, Zhang et al., 2020
**Drug metabolism and inactivation**	Upregulation of detoxifying enzymes and proteins (e.g., CYP450, GST, UGTs) leading to drug inactivation or reduced activation of prodrugs.	Direct blockade of the upregulated enzymes or delivery agents to destinated sites via protected manner.	Kaur et al., 2020, Pathania et al., 2018, Waarts et al., 2022
**Drug target mutation**	Mutations in drug targets (e.g., *EGFR*, *KRAS*) reduce drug binding affinity and efficacy, and activate related signaling pathways, leading to resistance to targeted therapies.	Adopt multi-modal blockade via next generation of selective inhibitors, protein degraders, or vertical/horizontal pathway combinations.	Huang et al., 2023, Du et al., 2025, Koga et al., 2021, Awad et al., 2021, Wang et al., 2025, Choucair et al., 2025
**Epigenetic regulation**	Involves DNA methylation, histone modifications, and non-coding RNAs (e.g., miRNAs, lncRNAs), which activate oncogenes, enhance DNA repair, and promote drug efflux.	Utilize epidrugs, including DNMTi, HDACi, or emerging ncRNA modulators to reprogram the resistance mechanism.	Sadida et al., 2024, Liu et al., 2020, Adhikari et al., 2022, He et al., 2020, Keith et al., 2011
**Hypoxia induction**	Promotes angiogenesis, reduces oxidative phosphorylation, enhances drug efflux, and induces a slow-cycling state, enabling survival under low oxygen conditions.	Combination with radiotherapy, glycolysis inhibitors, glutaminolysis inhibitors, and anti-VEGF agents.	Ortmann et al., 2024, Chen et al., 2023, Yong et al., 2022, Shang et al., 2025, Lv et al., 2015
**Protein damage response**	A cellular reaction triggered by anticancer drugs involves ubiquitination-mediated damage recognition, proteasome-dependent damage clearance, and mitophagy to remove drug-damaged mitochondria.	Proteasome inhibitors; E1 ligase inhibitors; autophagy inhibitors.	Shao et al., 2025, Li et al., 2025, Shao et al., 2020, Tang et al., 2025, Tang et al., 2025
**Tumor microenvironment (TME)**	Alteration of many components of TME, such as CAF, immune cells, blood & lymphatic vessels, ECM, and signaling molecules, is responsible for drug resistance.	Reagents that target various cellular components of TEM, angiogenesis, ECM, Immune checkpoint blockade, etc.	Wickramasekara et al., 2026, Boedtkjer et al., 2020, Dash et al., 2026, Chen et al., 2025

## The main characteristics of PDR and its effects on cancer therapy

Because protein damage occurs non-specifically before drugs act on their canonical targets (Shao et al., 2025), some critical questions arise: is protein damage a trivial side effect with minimal impact on cell viability; does PDR play a meaningful role in drug resistance; and what are the clinical applications of PDR? To address these, we describe key features and consequences of protein damage and PDR below.

### Vast majority of anticancer drugs cause ADPD

To comprehensively assess the prevalence of anticancer drug-induced protein damage, Shao et al. (2025) screened 101 FDA-approved anticancer drugs in MDA-MB-231 and A549 cells. Their data showed that ∼80% of drugs induced protein damage within 1 h, with further increases at 6 h. ADPD exhibits four defining characteristics: (i) Universality: this proteotoxic mechanism is not restricted to specific drug classes, affecting agents targeting DNA, tyrosine kinases, microtubules, and hormone receptors (Fig. 2A); (ii) diverse damage types: including protein oxidation, unfolding, misfolding, and aggregation (Fig. 2B); (iii) rapid onset: signs of protein misfolding are detectable within minutes (Fig. 2C), preceding canonical target effects (e.g., protein phosphorylation and DNA damage responses), typically manifesting hours later (Fig. 2D); and (iv) Preferentially target the newly synthesized proteins: experimental evidence using protein synthesis inhibitors (cycloheximide, puromycin) confirmed that ongoing protein synthesis is required for ADPD. Thus, anticancer drugs predominantly disrupt the folding of newly synthesized proteins, impairing their functions before maturation and proper subcellular localization.

**Figure 2.**
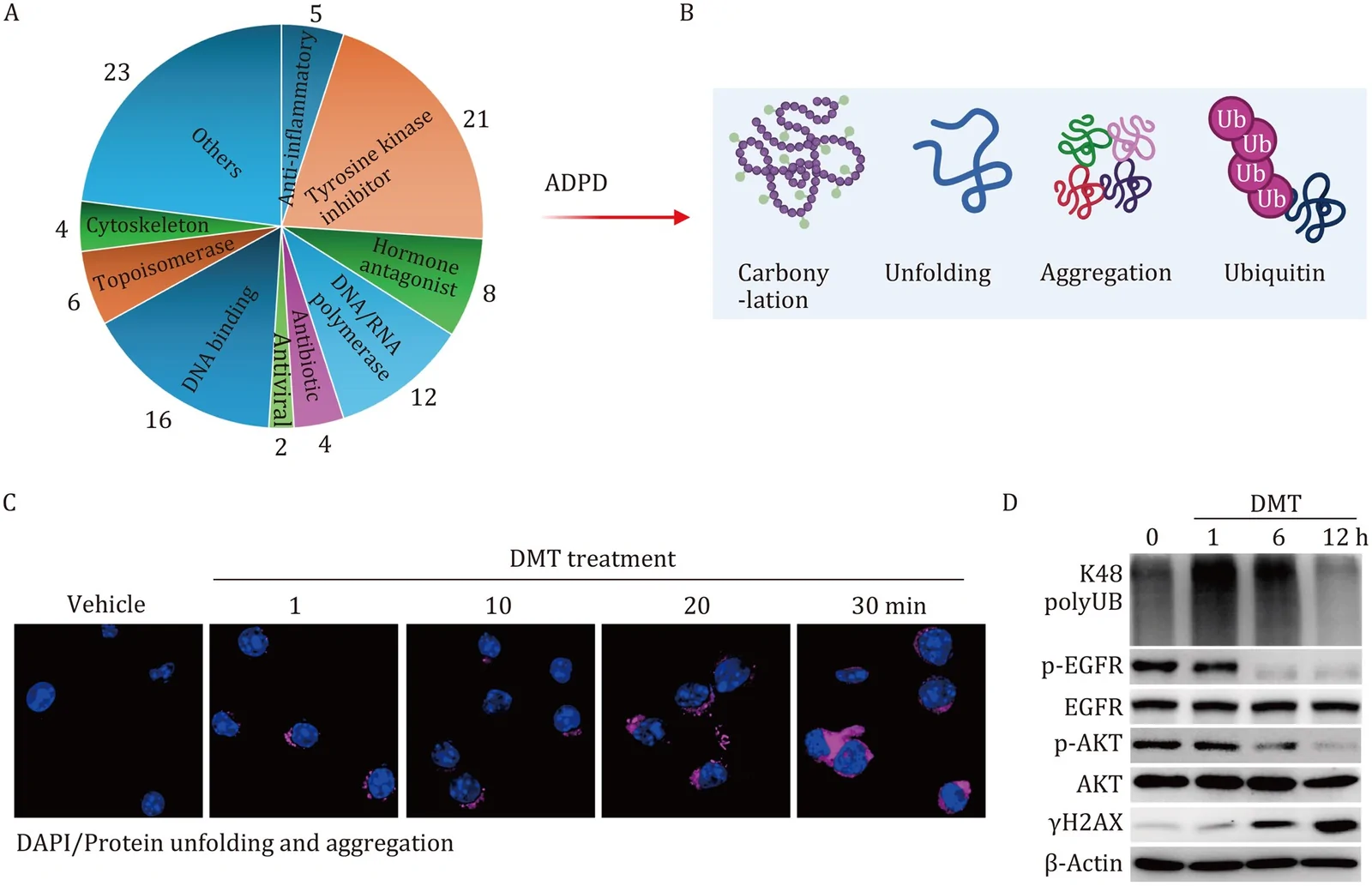
**Major features associated with ADPD**. (A and B) 101 anticancer drugs targeting DNA, DNA polymerase, microtubule, tyrosine kinase, topoisomerase, HDAC, etc. were tested for their ability to cause various types of acute drug protein damage (A), including protein oxidation, protein unfolding, and protein aggregation (B). The number of each different type of drugs were shown in the panel. (C and D) Anticancer drugs induce protein damage happen quickly and earlier than reaching their canonical target using dacomitinib (DMT), a selective inhibitor for the epidermal growth factor receptor (EGFR) (Peng et al., 2021), as an example. Apparently, DMT causes protein misfolding within a min upon treatment, while it effects on phosphorylation of EGFR and AKT as well as DNA damage hours later (D). Panel D is taken from Fig. 4j of Shao et al. (2025).

### Anticancer drugs trigger a ubiquitin-proteasome-dependent PDR

PDR is a finely tuned survival pathway consisting of damage recognition and clearance. Damage recognition centers on ubiquitination-mediated tagging of damaged proteins: following drug-induced protein damage, rapid K48-linked polyubiquitination marks proteins with a “degradation signal” (Draczkowski et al., 2025; Du et al., 2022; Manohar et al., 2019). Inhibition of the ubiquitin-activating E1 enzyme (via TAK-243) (Arakawa et al., 2024; Best et al., 2018) completely blocks this step, leading to unrecognized damaged protein accumulation and extensive aggregation. Damage clearance involves degradation of ubiquitinated proteins by the 26S proteasome—a critical process for maintaining cellular homeostasis and preventing toxic protein accumulation (Lee, 2025; Myeku and Duff, 2018; Sedlacek, 2025). Inhibition of proteasome activity (e.g., with bortezomib, BTZ) results in significant accumulation of K48-polyubiquitinated proteins, highlighting the proteasome’s essential role in executing PDR (Li et al., 2025; Shao et al., 2025).

PDR integrity is vital for cellular survival: inhibiting either recognition or clearance leads to overwhelming damaged protein accumulation, activating caspase-3 and accelerating cell death. Thus, ubiquitin-proteasome-dependent PDR acts as a robust protective buffer—its upregulation enables cancer cells to withstand anticancer drug-induced proteotoxic stress, promoting MDR (Li et al., 2025; Shao et al., 2025).

### The role of mitochondrion in drug-induced protein damage

Consistent with the role of the proteasome in moving damaged proteins, three cell lines, which were generated by graded increased concentration of cisplatin (from 1.5 µmol/L to 3 µmol/L and 10 µmol/L), had graded higher proteasome activity (Shao et al., 2020). Of note, the high proteasome activities were correlated with decreased mitochondrial respiratory activities and reduced levels of ATP that was reported to suppress proteasome activity (Geng et al., 2009; Huang et al., 2010; Majetschak, 2013). This observation raises a question regarding how the mitochondrion is damaged by the treatment of the drugs.

Mitochondria are uniquely susceptible to damage because ∼99% of their proteins are synthesized in the cytoplasm and imported in an unfolded state (Haastrup et al., 2023; Palmer et al., 2021). This makes nascent mitochondrial proteins particularly vulnerable to drug binding and damage during synthesis and import. Supporting this, proteomic analyses revealed significant enrichment of mitochondrial proteins—especially subunits of respiratory complexes I, II, and III—among cisplatin-bound and ubiquitinated proteins.

By tracing drug fluorescence, Li et al. (2025) recently demonstrated that chemical drugs (e.g., Cy5-labeled cisplatin) and auto-fluorescent drugs (e.g., mitoxantrone, doxorubicin, topotecan) are co-imported into mitochondria with mitochondrial proteins within minutes of treatment, in a protein synthesis-dependent manner. This co-import disrupts mitochondrial dynamics and reduces oxidative phosphorylation, leading to diminished ATP production.

### The coordination of mitochondrion and lysosome in PDR for mitophagy

Effectively targeted removal of damaged or surplus mitochondria via lysosomes—regulated by energy and environmental signals is essential for maintaining mitochondrial homeostasis (Wang et al., 2023; Wei et al., 2015). Li et al. (2025) demonstrated that chemical drugs induce ROS-BNIP3-mediated mitophagy, a process tightly coupled to proteasome activity. The proteasome suppresses mitochondrial import of damaged proteins and induces ROS-BNIP3-mediated mitophagy to remove damaged mitochondria (Li et al., 2025) (Fig. 3).

**Figure 3.**
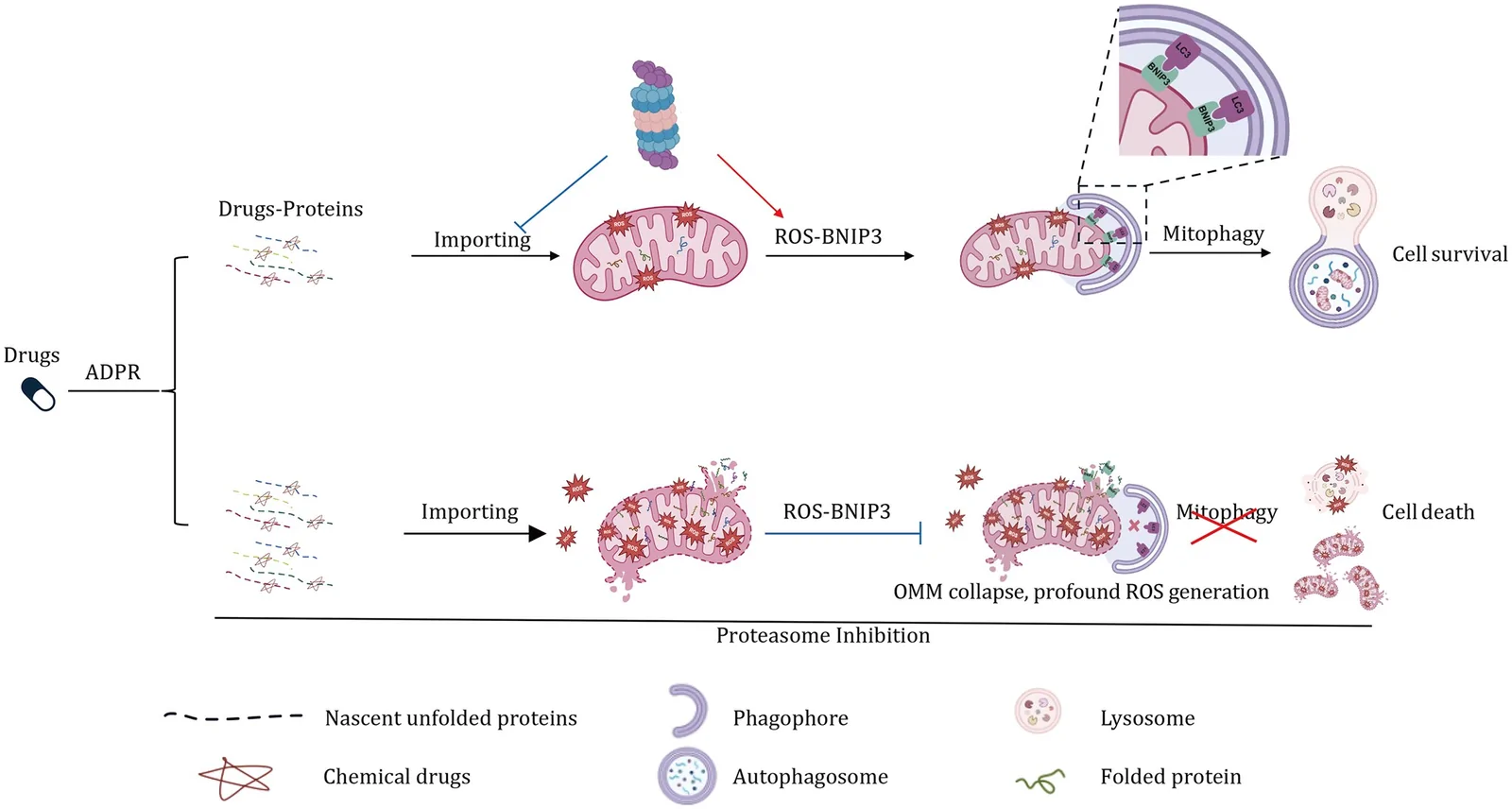
**Upon entering cells, chemical drugs interact nonspecifically with newly synthesized linear proteins, binding to amino acid residues and causing structural damage**. Under normal or elevated proteasome activity, only a limited number of damaged proteins is imported into mitochondria, leading to mitochondrial ROS (mtROS) production, which triggers protective mitophagy in an outer mitochondrial membrane (OMM)-localized, BNIP3 oxidation- and OMM integrity-dependent manner. However, the combination of chemical drugs and PI overwhelms mitochondrial capacity through uncontrolled import of damaged proteins, resulting in OMM collapse, ROS-related lysosomal membrane permeabilization, defective mitophagy, and irreversible proteostasis stress-induced cell death.

Key findings from this study include: (i) chemical drugs bind to newly synthesized yet unfolded mitochondrial proteins, which are co-imported into mitochondria before localizing to lysosomes and/or the nucleus; (ii) mitochondrial import of damaged proteins triggers a mitochondria-lysosome-mediated cascade, including mitochondrial integrity stress response (ISR), mitochondrial unfolded protein response (UPRmt), increased lysosome biogenesis, and PINK1-Parkin-independent, ROS-BNIP3-mediated mitophagy; (iii) proteasomes are central regulators of mitochondria-lysosome crosstalk, monitoring proteostasis, suppressing mitochondrial protein import, and promoting mitophagy under normal and drug-treated conditions; (iv) the proteasome inhibitor BTZ induces excessive mitochondrial import of damaged proteins, causing mitochondrial membrane breaks, massive mitochondrial ROS production and leakage, lysosome membrane permeabilization, and impaired mitophagy; and (v) combined BTZ and anticancer drug therapy induces a novel form of cell death by inhibiting mitophagy, overcoming tumor drug resistance.

## Proteasome activity as a new strategy to predict and overcome multidrug resistance

The pivotal role of PDR, particularly the increased proteasome activity in counteracting drug-induced cytotoxicity, offers a compelling strategy to predict and overcome MDR. Recent evidence from large patient databases (e.g., The Cancer Genome Atlas, TARGET) and clinical samples consistently shows that proteasome activity is significantly elevated in tumors compared to normal tissues, with further increases in malignant and metastatic lesions (Shao et al., 2025). Notably, breast and colon cancer patients with progressive disease (PD) following therapy exhibit markedly higher baseline proteasome activity than those with partial response (PR) or stable disease (SD), positioning proteasome activity as a robust MDR predictive biomarker (Shao et al., 2025).

These findings have significant translational implications: researchers have developed a highly sensitive kit to measure proteasome activity from micro-biopsy samples, enabling identification of patients with “proteasome-high” tumors likely to resist conventional therapies (Shao et al., 2025). This highlights proteasome inhibition as a rational approach to overcome MDR in high-risk patients. Preclinical models validate this strategy: in multidrug-resistant 231-R3 xenografts, combined cisplatin and carfilzomib (CFZ, a proteasome inhibitor) reversed resistance and significantly suppressed tumor growth. Moreover, in 3D tumor slice cultures (3D-TSCs) (Xing et al., 2021) and patient-derived organoids (PDOs) from treatment-resistant breast and colon cancer patients (Chen et al., 2021, 2025b), proteasome inhibitors (BTZ, CFZ, ixazomib [IXA]) restored sensitivity to diverse structurally unrelated anticancer agents (Shao et al., 2025). These findings were further corroborated in an investigator-initiated clinical trial: a breast cancer patient with high proteasome activity and progressive disease across multiple regimens achieved significant tumor reduction and clinical improvement following treatment with IXA plus gemcitabine.

In summary, the discovery of PDR reveals a common upstream mechanism underlying the action of many anticancer drugs and a unifying pathway for adaptive resistance. Measuring and therapeutically targeting proteasome activity enables personalized cancer therapy, effectively addressing the challenge of MDR (Fig. 4).

**Figure 4.**
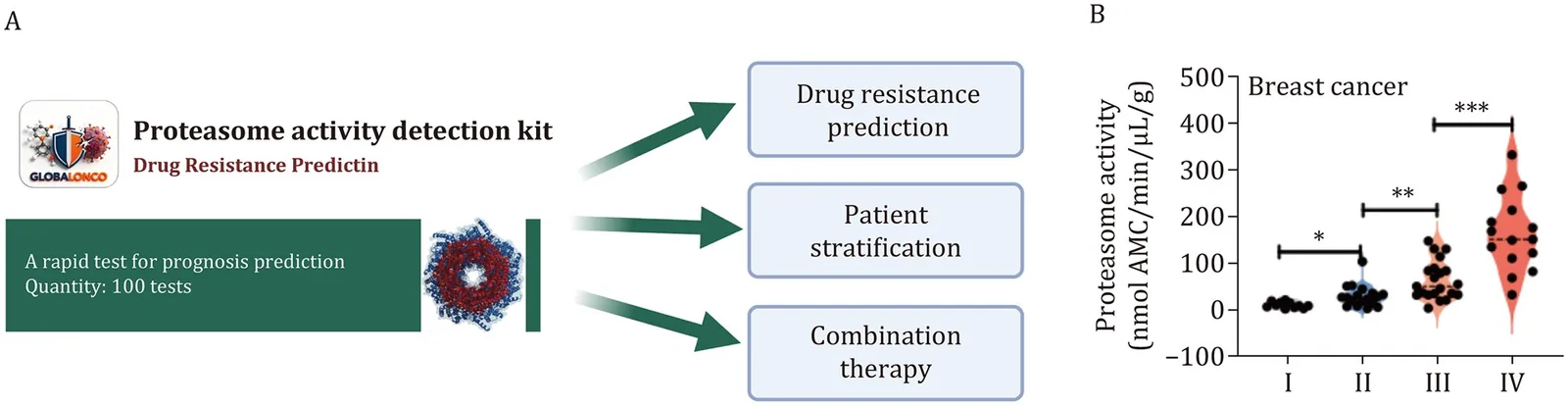
**Development of a highly sensitive kit for proteasome activity and its application to human breast cancer patients**. (A) A highly sensitive proteasome activity detection kit is capable of testing the proteasome activity of a little biopsy from tumor patients, which could be used to predict drug resistance, facilitating the patient stratification for further combination therapy. (B) The proteasome activity for breast cancer patients (*n *= 65) was detected by our kit, and proteasome activities in the indicated breast cancer tumor stages were compared. Panel B is taken from Fig. 6D of Shao et al. (2025).

## Strategies to enhance proteasome inhibitor efficacy for therapeutic treatment of solid cancers

Given the proteasome’s central role in mediating PDR and anticancer drug resistance, inhibiting proteasome activity is an attractive cancer therapeutic strategy. However, the three FDA-approved proteasome inhibitors (BTZ, CFZ, IXA) exhibit clinical efficacy primarily in liquid tumors, with limited effectiveness in solid tumors—prompting exploration of novel strategies to enhance their efficacy in solid tumor treatment (Chen et al., 2022; Fu et al., 2025; Rolfe, 2017; Zhang et al., 2022).

Combination therapies with proteasome inhibitors have shown promise. Our recent screen of over 100 anticancer drugs identified ammonium tetrathiomolybdate (TM) and AMD3100 as agents that increase the sensitivity of cancer cells, tumor slices, and patient-derived organoids to BTZ (Tang et al., 2025a, 2025b) (Fig. 5A). However, these combinations failed to inhibit solid tumor growth in nude mice likely due to insufficient *in vivo* drug concentrations, which explained the failure of many clinical trials where drugs poorly penetrate solid tumors (Fig. 5B). Notably, these drug combinations modulate signaling pathways to activate cytotoxic T cells, enhancing tumor killing in Balb/c mice, which have an intact immune background (Tang et al., 2025a). Furthermore, they induce antigen processing and presentation via the CCL5-MHC-I signaling cascade, facilitating CD8^+^ T cell activation and potent cancer cell elimination—amplifying the immune cycle and CD8^+^ T cell expansion (Fig. 5C). In *KRAS G12D*-mutation carrying mouse models and human patients, the combinations of HRS-4642 and CFZ inhibited tumor growth via enhancing interferon alpha response and reshaping tumor microenvironment to increase the number of IFN-γ and granzyme B positive CD8^+^ T cells (Tang et al., 2025a). In a preclinical cervical cancer model, BTZ combined with histone deacetylase inhibitor (Vorinostat) triggers the production of antigen-specific CD8^+^ T cells, stimulates the secretion of IFN-γ, and enhances killing effects of CD8^+^ T cells (Huang et al., 2015). These findings underscore the promising potential of combination therapies targeting proteasome activity in the treatment of solid tumors.

**Figure 5.**
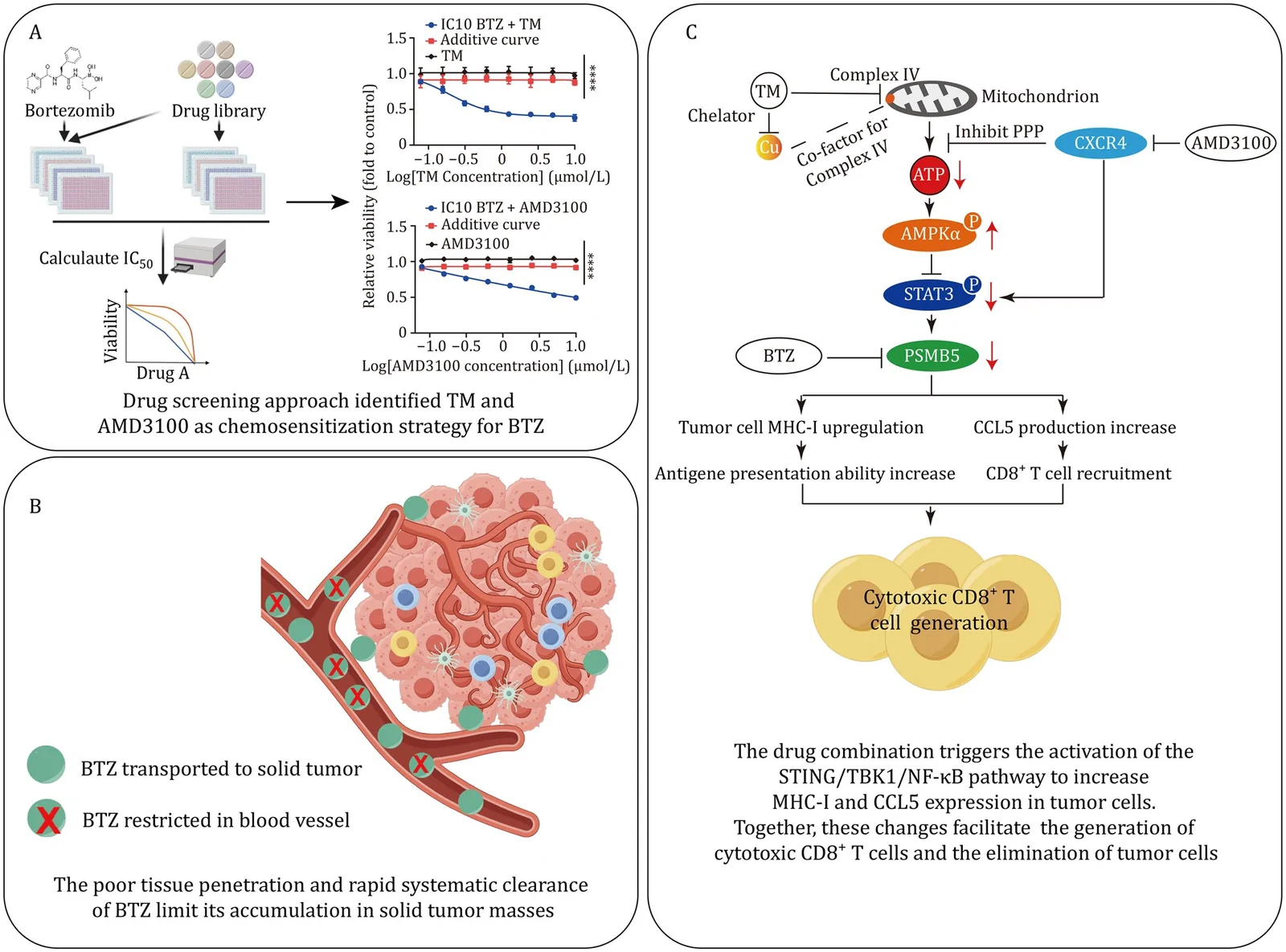
**Combination strategy to enhance the efficacy of BTZ**. (A) Drug combination screening identified TM and AMD3100 as chemosensitizing agents for BTZ. (B) Rapid clearance ability and poor accumulation capacity of BTZ in solid tumors led to the poor performance of BTZ against solid tumors. (C) TM inhibits oxidative phosphorylation, while AMD3100 inhibits the pentose phosphate pathway to reduce intercellular ATP level, activates AMPK, inhibits STAT3, and PSMB5 level, which increases the sensitivity of cancer cells to BTZ treatment. Drug combinations treatment activates the STING/TBK1/NF-κB pathway to increase the expression of MHC-I and CCL5, which together facilitate the generation of cytotoxic CD8^+^ T cells. MHC-I: major histocompatibility complex I, CCL5: chemokine (C-C motif) ligand 5. Panels A and C were adapted and updated from Tang et al. (2025a).

## Conclusion and future aspects

In this review, we have discussed in detail the acute drug protein damage (ADPD), which refers specifically to the swift protein damage caused by the vast majority of anticancer drugs and the PDR, a cellular action of cancer cells upon drug exposure (Fig. 5). PDR is a chain reaction involving ubiquitination-mediated damage recognition, proteasome-dependent damage clearance, and subsequent mitophagy for damaged mitochondria removal. Proteasome activity plays a critical role in PDR by clearing damaged proteins, inhibiting mitochondrial protein import, and promoting mitophagy (Li et al., 2025; Shao et al., 2020, 2025). Proteasome inhibitor-mediated PDR inhibition reverses the MDR of cancer cells by sensitizing them to anticancer drugs (Li et al., 2025; Shao et al., 2020, 2025). Following screening of over 100 anticancer drugs, Tang et al. (2025a) identified two specific combinations (TM + BTZ, AMD3100 + BTZ) that activate CD8^+^ T cells via inducing antigen processing and presentation through the CCL5-MHC-I signaling cascade (Fig. 6).

**Figure 6.**
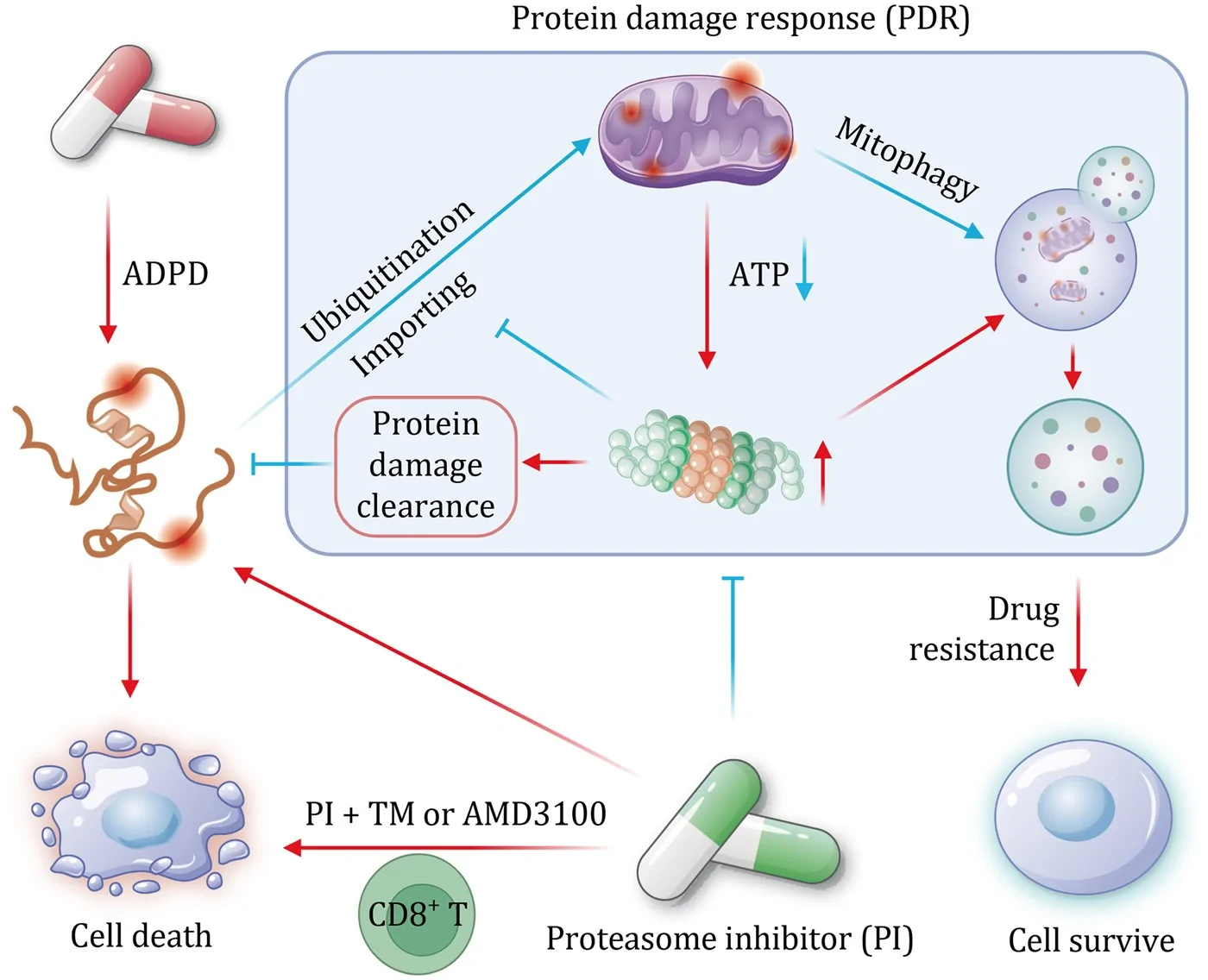
Illustration of key features of protein damage response (PDR) induced by anticancer drugs and the overcoming of MDR by inhibition of the PDR using proteasome inhibitor and the proper drug combinations.

Anticancer drug resistance occurs with high frequency and is the primary cause of treatment failure and high mortality in patients with late-stage cancer. Our discovery about ADPD and PDR may provide a promising strategy to determine the drug resistance status of cancer patients before, during and after treatment by measuring their proteasome activity, and thus, proper therapeutic options may be designed accordingly.

Because high proportions of the affected proteins are newly synthesized yet unfolded proteins, this suggests that the exposure of some specific amino acids might be the preferred binding sites. Currently, over 300 drugs have been approved by the FDA for cancer treatment; more drugs can also be tested for their ability to bind and damage proteins. Further efforts can also be directed to map the interaction sites between drugs and proteins. Li et al. (2025) found that treating cancer cells with BTZ markedly increased the accumulation of damaged proteins in mitochondria, highlighting a role of the proteasome system in preventing the importation of damaged proteins into the mitochondrion. While consistent with a ubiquitin-proteasome pathway for degrading damaged mitochondrial precursor proteins, the underlying mechanism, particularly the interplay between the proteasome system and mitochondrial membrane protein complexes/channels involved in protein import, remains elusive (Grevel and Becker, 2020; Hsu et al., 2025a; Yang et al., 2025).

Currently, the FDA has approved three proteasome inhibitors, BTZ, CFZ, and IXA, in the treatment of liquid cancers (Adams et al., 1999), while these inhibitors, in general, do not work well in clinical trials on solid cancers (Huang et al., 2014). As proteasome activity plays a critical role in modulating PDR, it is essential to enhance the efficacy of proteasome inhibition in centered therapies in solid cancers. In this aspect, the two drug combinations (i.e., TM/AMD3100 with BTZ) are promising (Tang et al., 2025a), but further studies are needed regarding how these drug combinations activate antitumor immunity and whether they can be used to treat some other types of solid cancers. Moreover, because none of the three proteasome inhibitors has been approved for the treatment of solid cancers so far, the application of these combinations to solid cancer might encounter potential clinical challenges. The ongoing efforts will be directed for enhancing therapeutic efficacy and examining potential side effect of the combination before conducting investigator initiated clinical study. Because the PI-based therapy may only be effective for patients with high levels of proteasome activities, Shao et al. (2025) developed a highly sensitive kit for measuring proteasome activities. The application of this kit should have a significant translational implication as it may enable the identification and subtyping of patients with high proteasome activity to support tailored therapeutic strategies. In the future, clinical trials with more patients may be identified before the personized treatment.

## Artificial intelligence (AI)

During the preparation of this work, the author(s) used Google Poe in order to check the grammar. After using this tool/service, the author(s) reviewed and edited the content as needed and take(s) full responsibility for the content of the publication.

## Abbreviations

5-FU: 5-fluorouracil
ABC: ATP-binding cassette
ADPD: acute drug protein damage
AML: acute myeloid leukemia
AMPK: AMP-activated protein kinase
AraC: cytarabine
ATPME: acidic tumor-permissive microenvironment
ATP7A: copper-transporting ATPase 1
BCRP: Breast Cancer Resistance Protein
BNIP3: BCL2 interacting protein 3
BRCA1/2: breast cancer associated gene 1/2
BTZ: bortezomib
CAFs: cancer-associated fibroblasts
CAR: chimeric antigen receptor
CCL5: chemokine (C-C motif) ligand 5
CDK6: cyclin-dependent kinase 6
CFZ: carfilzomib
CSCs: cancer stem cells
CYPs: cytochrome P450s
DDR: DNA damage repair
DMEs: drug-metabolizing enzymes
DNMT: DNA methyltransferase
ECM: extracellular matrix
EGFR: epidermal growth factor receptor
EMT: epithelial-mesenchymal transition
ER: estrogen receptor
FDA: Food and Drug Administration
GSTs: glutathione-S-transferases
HATs: histone acetyltransferases
HDACs: histone deacetylases
HIFs: hypoxia-inducible factors
HRR: homologous recombination repair
ICB: immune checkpoint blockade
ISR: integrity stress response
IXA: ixazomib
KRAS: Kirsten rat sarcoma viral oncogene homolog
LOXL2: lysyl oxidase-like 2
MDR: multidrug resistance
MDR1: multidrug resistance protein 1
MDSCs: myeloid-derived suppressor cells
MHC-I: major histocompatibility complex I
miRNAs: microRNAs
MRPs: Multidrug Resistance-Associated Proteins
mRCC: metastatic renal cell carcinoma
NBDs: nucleotide-binding domains
ncRNA: non-coding RNA
NER: nucleotide excision repair
NSCLC: non-small cell lung cancer
OCT4: octamer-binding transcription factor 4
OMM: outer mitochondrial membrane
PCD: programmed cell death
PD: progressive disease
PDOs: patient-derived organoids
PDR: protein damage response
P-gp: P-glycoprotein
PI: proteasome inhibitor
PR: partial response
RIPK3: receptor-interacting serine/threonine-protein kinase 3
ROS: reactive oxygen species
SD: stable disease
SLC: solute carrier
TAMs: tumor-associated macrophages
TGF-β: transforming growth factor beta
TKIs: , tyrosine kinase inhibitors
TM: , tetrathiomolybdate
TMDs: , transmembrane domains
TME: , tumor microenvironment
Tregs: , regulatory T cells
UGTs: , UDP-glucuronosyltransferases
ULK1: Unc-51 like autophagy activating kinase 1
UPRmt: mitochondrial unfolded protein response.
